# FSTL3 partially mediates the association of increased nonalcoholic fatty liver disease fibrosis risk with acute myocardial infarction in patients with type 2 diabetes mellitus

**DOI:** 10.1186/s12933-023-02024-x

**Published:** 2023-10-30

**Authors:** Wenfei Duan, Ruixiao Shi, Fang Yang, Zhoujunhao Zhou, Lihong Wang, Zhe Huang, Shufei Zang

**Affiliations:** 1 Department of Endocrinology, The Fifth People’s Hospital of Shanghai, Fudan University, 801 Heqing Road, Minhang District, Shanghai, 200240 China; 2 Department of Traditional Chinese Medicine, Maqiao Community Health Service Center, Minhang District, Shanghai, 20111 China; 3 Center of Community-Based Health Research, Fudan University, Shanghai, China; 4 Department of Genetics and Developmental Science, School of Life Sciences and Biotechnology, Shanghai Jiao Tong University, 800 Dongchuan Road, Shanghai, 200240 China

**Keywords:** Nonalcoholic fatty liver disease, Liver fibrosis, Type 2 diabetes mellitus, Acute myocardial infarction, Noninvasive biomarkers

## Abstract

**Background:**

The study aimed to investigate an association of increased liver fibrosis with acute myocardial infarction (AMI), and to investigate the mediating effect of serum follistatin-like protein 3 (FSTL3) on the association in patients with type 2 diabetes mellitus (T2DM).

**Method:**

A total of 1424 participants were included in this study, and were firstly divided into two groups: 429 T2DM patients and 995 T2DM patients with NAFLD to assess the association of NAFLD and AMI. Then 995 T2DM co-existent NAFLD patients were categorized by NAFLD fibrosis risk to explore the association between NAFLD fibrosis risk and AMI. Immunohistochemistry staining and semi-quantitative analysis of liver FSTL3 were performed in 60 patients with NAFLD. There were 323 individuals (191 without AMI and 132 with AMI) in T2DM co-existent NAFLD patients who had serum samples, and serum FSTL3 was tested and mediation effect of FSTL3 in association of NAFLD fibrosis and AMI was performed.

**Results:**

First, increased NAFLD fibrosis risk was an independent risk factor for AMI in patients with T2DM and co-existent NAFLD. In addition, analysis of Gene Expression Omnibus (GEO) database and immunohistochemical staining confirmed the increased expression of FSTL3 in the liver of NAFLD patients with fibrosis. Serum FSTL3 significantly increased in patients with high NAFLD fibrosis risk and AMI, and closely associated with NAFLD fibrosis and AMI severity in T2DM patients with co-existent NAFLD. Most importantly, analysis of the level of mediation revealed that increased serum FSTL3 partially mediated the association of increased NAFLD fibrosis risk with AMI in T2DM patients with co-existent NAFLD.

**Conclusions:**

NAFLD fibrosis was closely associated with AMI in T2DM patients. FSTL3 expression was enriched in the liver of NAFLD patients with significant and advanced fibrosis, and serum FSTL3 partially mediated the association of increased liver fibrosis risk with AMI in T2DM patients.

**Supplementary Information:**

The online version contains supplementary material available at 10.1186/s12933-023-02024-x.

## Introduction

Nonalcoholic fatty liver disease (NAFLD) is one of the most common chronic liver diseases worldwide. It comprises a spectrum of liver abnormalities ranging from a nonalcoholic fatty liver (NAFL) to nonalcoholic steatohepatitis (NASH) with or without fibrosis, and may progress to cirrhosis and hepatocellular carcinoma [1]. Further more, NAFLD may be complicated with extra hepatic cancers such as colorectal cancer, thyroid cancer, genitourinary system tumors and bladder cancer, etc. [2, 3]. As a liver manifestation of metabolic syndrome, NAFLD poses a similar cardiometabolic risk to coronary artery disease (CAD) including inflammation, dyslipidemia and endothelial dysfunction. There is mounting evidence that NAFLD is independently related to coronary artery disease [4, 5].

Acute myocardial infarction (AMI) is the most serious clinical manifestation of CAD and the most common cause of death in NAFLD patients due to acute coronary artery occlusion [6, 7]. Previous studies showed that NAFLD increases the risk of myocardial infarction [8, 9]. At present, type 2 diabetes mellitus (T2DM) usually co-existent with NAFLD and metabolic syndrome (MetS) [10] prompting a need to explore the relationship between NAFLD fibrosis and AMI in patients with T2DM.

Previous studies in patients with T2DM showed that NAFLD, as determined by liver ultrasound, was associated with an increased risk of cardiovascular events [11, 12]. Recent studies showed that both liver steatosis and fibrosis are associated with cardiovascular diseases [13–15]. Nonetheless other researchers report that significant fibrosis, detected by FibroScan, not liver steatosis, was closely associated with the occurrence of AMI in T2DM patients [13, 16]. It is unclear whether the presence of NAFLD or fibrosis confers an additional risk of developing CAD in patients with T2DM. In addition, the molecular mechanism underlying the association of NAFLD/fibrosis with AMI has not been fully illustrated.

Follistatin-like protein 3 (FSTL3) is a lipoendocrine factor that is found in various tissues such as placenta, heart and liver [17]. It mainly binds to members of the transforming growth factor β (TGF-β) superfamily such as activin A and myostatin to inhibit their bioactivity [18]. FSTL3 has been found to regulate glucose and lipid metabolism in mice [19]. Clinical studies have shown that serum FSTL3 level is higher in patients with NASH than in those with NAFL, even after adjusting for body mass index (BMI), age and sex [20]. In addition, peripheral FSTL3 level and cardiac FSTL3 level have been found to be related to coronary atherosclerosis and AMI, respectively [21, 22]. Koplev et al. revealed that FSTL3 is associated with atherosclerosis, and injection of recombinant FSTL3 protein could affect triglyceride content in the liver [23]. Nonetheless in patients with co-existent T2DM and NAFLD, the relationship between FSTL3 and AMI is unclear.

The purposes of this study are as follows: firstly, to investigate the association of NAFLD and its fibrosis risk with occurrence and severity of AMI in patients with T2DM; and secondly, to determine the mediating role of serum FSTL3 in the association of increased NAFLD fibrosis risk with occurrence and severity of AMI.

## Materials and methods

### Participants

**Fig. 1:**
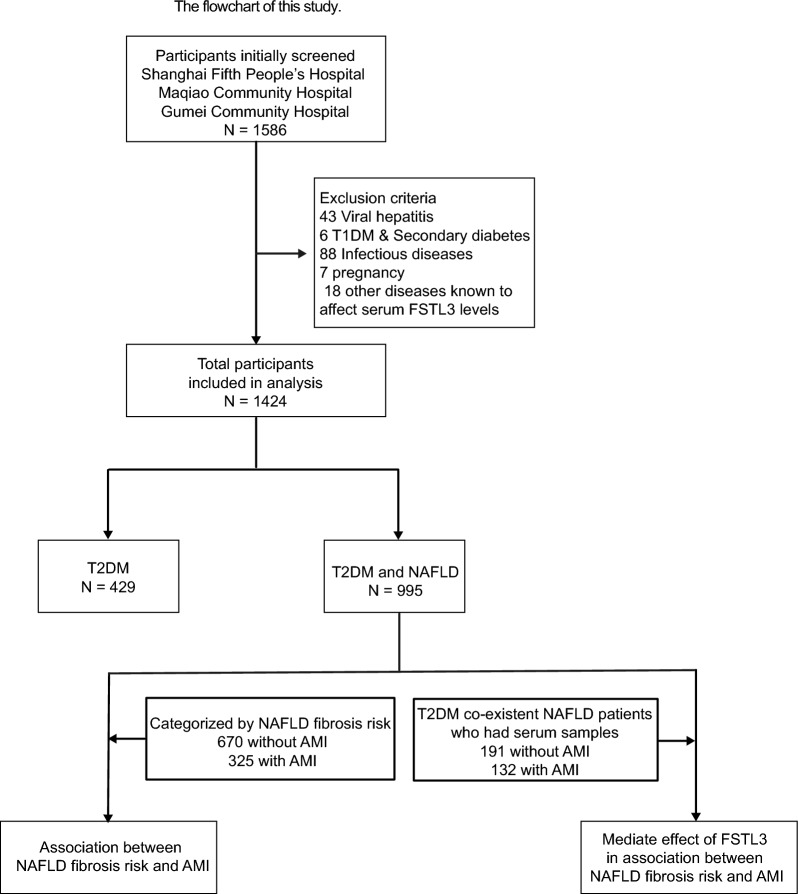
Flow chart of study

From January 2017 to December 2022, a total of 1586 participants participated in the study at Shanghai Fifth People’s Hospital Fudan University, Maqiao and Gumei Community Hospital in Minhang District of Shanghai. The study process is shown in Fig. 1. The diagnosis of type 2 diabetes mellitus was according to the 2017 criteria of the Chinese Diabetes Association: (1) Typical symptoms of diabetes (polydipsia, polyuria, polydipsia, weight loss) plus (1) random blood glucose ≥ 11.1 mmol/L; or (2) fasting blood glucose ≥ 7.0 mmol/L; or (3) 2 h blood glucose for oral glucose tolerance test ≥ 11.1 mmol/L. NAFLD was dignosed based on the same criteria: echogenicity of the liver significantly increased relative to that of the kidneys, the ultrasound beam was attenuated with the diaphragm indistinct, or the echogenic walls of the portal veins were less visible [24] rather than biopsy due to the easy availability and affordability of the former (Machine model of ultrasound instruments in the three hospitals we selected are all GE LOGIQ series which ensures the comparability of ultrasonography results to the greatest extent and all the utrasound staff are uniformly trained). The diagnosis of AMI was based on the criteria set by the European Heart Association (ESC) in 2012: The cardiac biomarker hypersensitive troponin (hs-cTnT or I) increases and / or decreases, and at least one value is higher than the 99th percentile of the reference upper limit, and at least one of the following criteria was met: (1) symptoms of myocardial ischemia; (2) new or presumed new significant ST-T changes or newly developed left vertical bundle branch block (LBBB); (3) pathological Q wave appeared on ECG; (4) imaging examination showed viable myocardial loss or new abnormal wall motion consistent with ischemic etiology; (5) coronary artery thrombosis confirmed by angiographic examination or autopsy.

A total of 1424 participants were included in the final analysis, and were firstly divided into two groups: 429 T2DM patients and 995 T2DM patients with NAFLD to assess the association of NAFLD and AMI. Then 995 T2DM co-existent NAFLD patients were categorized by NAFLD fibrosis risk to explore the association between NAFLD fibrosis risk and AMI, among which 323 individuals (191 without AMI collected from the Department of Endocrinology in Fifth People’s Hospital of Shanghai and 132 with AMI collected from the Metabolic Disease and Stroke and AMI Nursing Center) in 995 T2DM co-existent NAFLD patients had serum samples, then serum FSTL3 were measured and mediation analysis were performed.

Patients were excluded if they had: (1) viral hepatitis such as autoimmune hepatitis, alcoholic liver disease or other liver diseases caused by toxicity; (2) type 1 diabetes or other specific types of diabetes; (3) a history of acute or chronic infectious disease in the two weeks prior to blood testing; (3) pregnancy; (4) other diseases known to affect serum FSTL3 level such as eclampsia, abortion, gastric or kidney cancer.

This study was approved by the Ethics Committee of Shanghai Fifth People’s Hospital Fudan University. Informed consent was obtained from patients or their representatives, and the protocol conformed with the Declaration of Helsinki.

### Data collection and laboratory measurements

Height, weight, waist circumference (WC), and blood pressure of all patients were measured according to standard protocols. Body mass index (BMI) was calculated by dividing weight in kilograms by height in meters squared. Venous blood samples were obtained after an overnight fast (at least 10 h) and biochemical parameters (Automatic Biochemical Analyzer, Roche Cobas 8000) and blood cell count (Automatic blood cell analyzer, Sysmex XN 9000) measured. Fasting blood glucose (FBG), glycated hemoglobin (HBA_1_C), total cholesterol (TC), triglycerides (TGs), high density lipoprotein cholesterol (HDL-C), low density lipoprotein cholesterol (LDL-C), alanine aminotransferase (ALT), aspartate aminotransferase (AST), total protein (TP), serum albumin (ALB), globulin (GLB), glutamine transpeptide (γ-GT), white blood cell count (WBC), red blood cell count (RBC), hemoglobin (HGB), platelet (PLT), total bilirubin (TBIL), direct bilirulin (DBIL), serum creatinine (Scr), uric acid (UA), blood urea nitrogen (BUN), C reactive protein (CRP), cardiac troponin I (cTnI) and estimate glomerular filtration rate (eGFR) were measured.

### Measurement of serum FSTL3 concentration

Whole blood (1 mL) was collected from each patient and serum obtained to measure FSTL3 concentration using an enzyme-linked immunosorbent assay (ELISA) kit purchased from R&D Systems (#DFLRGO) according to the manufacturer’s instructions.

### Semi-quantitative analysis of immunohistochemistry staining

A semi-quantitative analysis of formalin paraffin-embedded liver biopsy specimens was performed in 30 NAFLD patients with significant and advanced fibrosis and 30 non-NAFLD patients using FSTL3 immunohistochemical staining. The antibody was purchased from Sigma (no. HPA045378, 1:40). Image J was used to observe the results under 20 times amplification and average FSTL3 positive area in each section converted to the average optical density (AOD) before comparation.

### Fibrosis risk in nonalcoholic fatty liver disease

NFS was calculated according to the published formula NFS =  − 1.675 + 0.037 × age (years) + 0.094 × BMI (kg/m^2^) + 1.13 × impaired fasting glucose or diabetes (yes = 1, no = 0) + 0.99 × AST/ALT− 0.013 × PLT count (× 10^9^/L)− 0.66 × serum albumin (g/dL). Two cut-off values were used to divide NAFLD patients into three groups: low risk group (NFS <− 1.455), medium risk (NFS: − 1.455–0.676) and high risk (NFS > 0.676 or > 65 years old, NFS > 0.12) [25]. The FIB-4 index was calculated according to the formula FIB-4 index = age (years) × AST (U/L)/(PLT(× 10^9^/L) × (ALT (U/L))^1/2^). Two cut-off values were applied to divide NAFLD patients into three groups: low risk group (FIB-4 < 1.30), medium risk (FIB-4: 1.30–3.25) and high risk (FIB-4 > 3.25 or > 65 years old, FIB4 > 2.0) [25].

### Gensini score

An ACC/AHA scoring method was adopted to generate the Gensini score [26] (score was performed at the most severe stenosis site): stenosis ≤ 25% 1 point; stenosis 26 to 50% 2 points; stenosis 51 to 75% 4 points; stenosis 76 to 90% 8 points; stenosis 91 to 99% 16 points; stenosis 100% 32 points. Coronary artery score (CAS) of each vessel was calculated as the stenosis score × weight coefficient of the vessel. The Gensini score was the sum of all vascular scores.

### Statistical analysis

Baseline characteristics were analyzed by the frequency of categorical variables and the mean ± SD or median (quartile range) of continuous variables. Categorical variables were analyzed by chi-square test. All continuous variables were tested for normality and then t tests/analysis of variance or nonparametric tests used to determine inter-group differences. Pearson correlation analysis was used to evaluate the correlation between noninvasive hepatic fibrosis score NFS/FIB-4 and Gensini score and other indicators. Logistic regression analysis was used to determine the association between NFS/FIB-4 and the risk of AMI. Spearman correlation was used to analyze the correlation between serum FSTL3 concentration and NFS/FIB-4 and Gensini score and other indicators in T2DM patients with co-existent NAFLD. Analysis of degree of mediation was performed to demonstrate the effect of serum FSTL3 on the association of increased fibrosis risk and AMI in T2DM patients. The mediating effect percentage was evaluated by R function. The main parameter was the proportion of mediation, and based on the formula: (indirect effect/total effect) × 100%. All statistical analyses were performed using SPSS 27.0 software (IBM SPSS Inc) and R (version 4.0.5, R Foundation for Statistical Computing). All p values were two-tailed (p < 0.05).

## Results

### NAFLD fibrosis risk was closely associated with AMI in patients with T2DM

Among 1429 individuals, although patients with T2DM co-existent with NAFLD had a higher prevalence of artery hypertension, waist circumference, BMI, DBP, increased level of FBG, lipids (TG, TC, LDL-C), liver enzymes (ALT, AST), TP, ALB, prealbumin, γ-GT, WBC, lymphocyte (%), lymphocyte, eosinophilic granulocyte (%), basophilic granulocyte (%), RBC, HGB, TBIL, UA and eGFR compared with those without NAFLD, there was no significant difference in the prevalence of AMI between the two groups (32.7% *vs* 33.1%, p = 0.872) (Additional file 1: Table S1). Logistic regression analysis showed that NAFLD was not an independent risk factor for AMI (Additional file 1: Table S2).

When 995 patients with T2DM and NAFLD were divided into low, intermediate and high fibrosis risk subgroups based on NFS, there was a stepwise increase in the prevalence of AMI with increasing NAFLD fibrosis risk (13.86% *vs* 28.05% *vs* 50.92%, p < 0.001) (Table 1). When stratified by FIB-4, the prevalence of AMI also increased in a stepwise manner from low NAFLD fibrosis risk to high fibrosis risk (20.20% *vs* 30.74% *vs* 63.59%, p < 0.001) (Table 1). Subsequently, T2DM patients with co-existent NAFLD who had AMI were then divided into low-to-intermediate risk and high risk subgroups on the basis of NFS (Table 2). Gensini scores were much higher in patients with high fibrosis risk group than patients with low-to-intermediate risk (55.0 [31.0, 80.0] vs 42.0 [26.5, 62.5], p = 0.036). When patients were divided into low-to- intermediate risk and high-risk subgroups according to FIB-4, Gensini score was also much higher in patients with high fibrosis risk than patients with low-to-intermediate risk (58.0 [32.0, 90.0] vs 42.0 [27.0, 62.2], p < 0.001). Pearson correlation analysis showed a significant positive correlation between NFS/FIB-4 and Gensini score in T2DM patients with co-existent NAFLD and AMI (Additional file 1: Table S3).

**Table 1:** Comparison of parameters among different NAFLD fibrosis risk stages stratified according to NFS and FIB-4

		Total participates (n = 995)			Total participates (n = 995)			
	NFS-LR	NFS-IR	NFS-HR	*P* value	FIB4-LR	FIB4-IR	FIB4-HR	*P* value
N (%)	202 (20.30%)	467 (46.94%)	326 (32.76%)		495 (49.75%)	283 (28.44%)	217 (21.81%)	
Gender, male (n/%)	127 (62.87%)	299 (64.03%)	185 (56.75%)	0.104	318 (64.24%)	170 (60.07%)	123 (56.68%)	0.140
Age (Year)	50.0 (42.3, 58.0)	61.0 (54.0, 66.0)^b^	70.0 (66.0, 77.0)^bd^	** < 0.001**	57.5 (48.0, 65.0)	64.0 (59.0, 70.0)^b^	71.0 (65.0, 77.0)^bd^	** < 0.001**
Smoking (n/%)	82 (40.59%)	203 (43.47%)	138 (42.33%)	0.785	207 (41.82%)	121 (42.76%)	95 (43.78%)	0.884
Arterial hypertension (n/%)	96 (47.52%)	253 (54.18%)	200 (61.35%)	**0.007**	250 (50.51%)	157 (55.48%)	142 (65.44%)	** < 0.001**
waist circumference (cm)	92 (86, 100)	94 (87, 103)	94 (88, 102)	0.099	94 (87, 104)	94 (88, 101)	93 (87, 99)	0.228
BMI (Kg/m^2^)	25.59 (24.01, 27.75)	26.67 (24.69, 28.85)	26.42 (24.54, 28.73)	0.121	26.84 (24.69, 29.30)	26.29 (24.61, 28.40)	26.18 (23.80, 28.09)^a^	**0.009**
Duration of diabetes (Year)	5 (2, 10)	9 (4, 14)^b^	10 (5, 19)^bd^	** < 0.001**	8 (3, 13)	10 (5, 14)^a^	10 (5, 18)^b^	** < 0.001**
SBP (mmHg)	130 (118, 140)	130 (120, 141)	135 (122, 145)^bc^	** < 0.001**	130 (120, 141)	135 (121, 145)^b^	133 (120, 142)	**0.003**
DBP (mmHg)	80 (72, 88)	80 (70, 88)	77 (70, 84)^bd^	** < 0.001**	80 (71, 89)	80 (70, 86)	75 (70, 82)^bc^	** < 0.001**
FBG (mmol/L)	8.5 (6.6, 10.9)	7.6 (6.1, 9.6)^b^	7.7 (5.9, 10.1)^a^	**0.004**	7.8 (6.0, 10.1)	7.6 (6.0, 9.5)	8.3 (6.3, 10.8)	0.325
HbA_1_C (%)	9.0 (7.5, 10.7)	8.3 (7.0, 9.9)	8.4 (7.2, 10.2)^b^	**0.031**	8.7 (7.1, 10.2)	8.3 (7.3, 10.1)	8.0 (7.1, 10.1)^b^	**0.009**
TC (mmol/L)	4.64 (3.80, 5.48)	4.32 (3.42, 4.97)^b^	3.87 (3.21, 4.77)^bd^	** < 0.001**	4.42 (3.54, 5.11)	3.97 (3.32, 4.97)^a^	4.00 (3.19, 4.76)^b^	** < 0.001**
TG (mmol/L)	2.05 (1.45, 3.00)	1.73 (1.28, 2.68)^a^	1.55 (1.10, 2.27)^bc^	** < 0.001**	1.81 (1.33, 2.87)	1.67 (1.14, 2.45)	1.58 (1.13, 2.25)^b^	**0.003**
HDL-C (mmol/L)	0.96 (0.81, 1.13)	0.94 (0.80, 1.13)	0.99 (0.83, 1.19)	0.297	0.93 (0.80, 1.13)	0.99 (0.82, 1.16)	0.99 (0.84, 1.17)^a^	**0.033**
LDL-C (mmol/L)	2.91 (2.19, 3.77)	2.70 (1.97, 3.44)	2.49 (1.77, 3.17)^bc^	** < 0.001**	2.79 (2.03, 3.50)	2.55 (1.84, 3.26)^a^	2.43 (1.80, 3.25)^b^	** < 0.001**
ALT (μ/L)	27.0 (18.2, 41.6)	20.3 (14.6, 34.0)^a^	19.0 (13.0, 30.5)^b^	** < 0.001**	20.0 (14.1, 29.9)	19.5 (13.9, 34.0)	27.0 (16.7, 39.9)^bc^	** < 0.001**
AST (μ/L)	18.0 (14.0, 24.8)	17.2 (13.8, 26.0)	20.8 (15.0, 38.6)^bd^	** < 0.001**	16.0 (13.0, 21.0)	19.0 (14.6, 28.5)^b^	31.1 (20.3, 102.1)^bd^	** < 0.001**
Total protein (g/L)	69.8 ± 6.4	68.2 ± 6.2^b^	65.8 ± 6.2^bd^	** < 0.001**	68.5 (65.0, 73.9)	70.0 (65.1, 72.6)	65.9 (61.4, 70.5)^bd^	** < 0.001**
Albumin (g/L)	45.4 (42.9, 48.2)	43.0 (41.0, 46.6)^b^	40.3 (38.2, 43.0)^bd^	** < 0.001**	43.6 (41.0, 46.8)	42.4 (40.0, 46.0)^a^	40.0 (38.3, 43.0)^bd^	** < 0.001**
Prealbumin (g/L)	0.28 (0.23, 0.32)	0.25 (0.22, 0.28)^b^	0.23 (0.19, 0.25)^bd^	** < 0.001**	0.26 (0.22, 0.29)	0.24 (0.22, 0.27)^a^	0.21 (0.17, 0.25)^bd^	** < 0.001**
Globulin (g/L)	23.8 ± 5.06	24.7 ± 4.3	25.1 ± 5.1^a^	0.121	24.8 (21.5, 28.4)	25.0 (22.2, 27.4)	25.9 (22.9, 28.3)	0.075
γ-GT (μ/L)	29.5 (22.0, 43.8)	28.0 (19.0, 43.8)	25.0 (19.0, 38.0)^b^	**0.005**	26.0 (20.0, 41.0)	28.5 (19.0, 41.0)	29.0 (21.0, 51.2)^b^	0.720
WBC (× 10^9^/L)	6.89 (5.61, 8.29)	6.64 (5.66, 7.57)	6.38 (5.14, 8.39)	0.056	6.72 (5.71, 7.88)	6.45 (5.50, 7.44)^a^	6.35 (4.92, 9.09)	**0.024**
Neutrophil (%)	57.4 ± 8.9	60.2 ± 8.9^b^	63.4 ± 11.5^bd^	** < 0.001**	60.0 (53.6, 65.9)	59.8 (54.2, 64.8)	65.8 (56.5, 75.1)^bd^	** < 0.001**
Neutrophil (× 10^9^/L)	4.13 (3.05, 5.08)	3.86 (3.16, 4.83)	3.97 (2.91, 5.76)	0.282	4.02 (3.27, 5.00)	3.61 (3.00, 4.57)^a^	4.02 (2.75, 6.87)^d^	**0.001**
Lymphocyte (%)	31.95 (26.95, 36.60)	28.35 (24.13, 35.38)^b^	27.15 (19.33, 32.60)^bd^	** < 0.001**	29.50 (24.83, 35.48)	28.80 (24.58, 34.60)	23.80 (15.70, 32.25)^bd^	** < 0.001**
Lymphocyte (× 10^9^/L)	2.11 (1.74, 2.66)	1.92 (1.48, 2.35)^b^	1.63 (1.31, 2.04)^bd^	** < 0.001**	1.99 (1.65, 2.41)	1.82 (1.42, 2.35)^b^	1.49 (1.15, 1.92)^bd^	** < 0.001**
Neutrophil/ lymphocyte	1.81 (1.45, 2.39)	2.13 (1.51, 2.72)^a^	2.33 (1.73, 3.69)^bd^	** < 0.001**	2.04 (1.52, 2.69)	2.01 (1.61, 2.57)	2.79 (1.77, 4.92)^bd^	** < 0.001**
Eosinophilic granulocyte (%)	2.0 (1.2, 2.8)	2.0 (1.3, 3.2)	2.2 (1.4, 3.3)	0.440	1.6 (1.2, 2.9)	2.3 (1.6, 3.5)^b^	2.3 (1.6, 3.3)	**0.005**
Basophilic granulocyte (%)	0.4 (0.3, 0.5)	0.4 (0.2, 0.6)	0.4 (0.2, 0.5)	0.140	0.4 (0.2, 0.5)	0.4 (0.2, 0.6)	0.3 (0.2, 0.5)	0.630
Mononuclear leucocyte (%)	6.6 (5.2, 8.1)	6.8 (5.8, 7.9)	7.4 (5.9, 8.7)^a^	**0.013**	6.6 (5.3, 7.7)	6.9 (5.9, 8.2)	7.6 (6.4, 8.4)^a^	**0.012**
PLT (× 10^9^/L)	266 (245, 314)	209 (177, 241)^b^	168 (138, 197)^bd^	** < 0.001**	237 (205, 272)	173 (152, 204)^b^	162 (128, 197)^b^	** < 0.001**
RBC (× 10^12^/L)	4.9 (4.5, 5.3)	4.7 (4.3, 5.0)^b^	4.4 (3.9, 4.5)^bd^	** < 0.001**	4.7 ± 0.5	4.5 ± 0.6^b^	4.1 ± 0.6^bd^	** < 0.001**
HGB (g/L)	144 (135, 159)	141 (128., 151)^b^	129 (120, 140)^bd^	** < 0.001**	143 (131, 152)	132 (124, 147)^b^	126 (114, 136)^b^	** < 0.001**
TBIL (mmol/L)	9.5 (7.2, 12.2)	10.0 (7.4, 14.0)	9.8 (7.2, 13.0)	0.309	9.6 (7.3, 13.7)	10.3 (6.7, 13.9)	9.4 (7.6, 11.6)^bc^	**0.003**
DBIL (mmol/L)	3.6 (2.8, 4.3)	3.8 (3.0, 5.0)	3.8 (3.0, 5.2)	0.081	3.5 (2.8, 4.6)	3.5 (2.7, 4.9)	3.7 (3.3, 4.3)^bd^	** < 0.001**
Scr (μmol/L)	60.0 (49.0, 75.8)	66.0 (56.0, 80.5)^b^	74.5 (58.3, 95.8)^bd^	** < 0.001**	65.0 (55.0, 78.5)	71.0 (58.0, 89.0)	62.0 (54.0, 83.0)^a^	**0.007**
UA (μmol/L)	329.5 (267.5, 404.8)	336.5 (275.3, 390.0)	332.0 (276.0, 400.5)	0.497	331.0 (277.0, 395.0)	340.5 (275.3, 393.0)	324.0 (257.0, 393.0)	0.510
BUN (mmol/L)	5.0 (4.2, 5.8)	5.3 (4.4, 6.5)^a^	5.7 (4.7, 7.5)^bd^	** < 0.001**	5.1 (4.4, 6.3)	5.6 (4.5, 6.9)^b^	5.4 (4.5, 7.5)^b^	** < 0.001**
C reactive protein (mg/dl)	1.0 (1.0, 3.0)	1.0 (1.0, 3.0)	1.0 (1.0, 2.0)	0.508	1.0 (1.0, 3.0)	1.0 (1.0, 3.0)	1.0 (1.0, 2.0)	0.604
cTnI (ng/ml)	0.01 (0.01, 0.02)	0.01 (0.01, 0.02)	0.03 (0.01, 7.39)^bd^	** < 0.001**	0.01 (0.01, 0.02)	0.01 (0.01, 0.08)^a^	0.76 (0.02, 10.35)^bd^	** < 0.001**
eGFR (ml/min)	106 (92, 116)	94 (78, 103)^b^	85 (60, 94)^bd^	** < 0.001**	98 (83, 109)	90 (71, 100)^b^	85 (63, 96)^b^	** < 0.001**
AMI (n/%)	28 (13.86%)	131 (28.05%)	166 (50.92%)	** < 0.001**	100 (20.20%)	87 (30.74%)	138 (63.59%)	** < 0.001**

Bold P values represent P < 0.05

^a^VS NFS/FIB-4 LR: p < 0.05

^b^VS NFS/FIB-4 LR: p < 0.01

^c^VS NFS/FIB-4 IR: p < 0.05

^d^VS NFS/FIB-4 IR: p < 0.01

**Table 2:** Comparison of parameters of different NAFLD fibrosis risk stages in patients with acute myocardial infarction

	Total participates (n = 325)			Total participates (n = 325)		
	NFS-LR-IR	NFS-HR	P value	FIB4-LR-IR	FIB4-HR	P value
N (%)	159 (48.92%)	166 (51.08%)		187 (57.54%)	138 (42.46%)	
Gender, male (n/%)	105 (66.04%)	110 (66.27%)	0.965	145 (77.54%)	108 (78.26%)	0.877
Age (Year)	63.0 (55.0, 68.5)	71.0 (66.0, 77.0)	** < 0.001**	64.5 (58.0, 69.3)	71.0 (63.0, 77.0)	** < 0.001**
Smoking (n/%)	90 (56.60%)	113 (68.07%)	**0.033**	110 (58.82%)	97 (70.29%)	**0.034**
Arterial hypertension (n/%)	100 (62.89%)	124 (74.70%)	**0.022**	131 (70.05%)	111 (80.43%)	**0.034**
waist circumference (cm)	93.3 (87.9, 97.4)	93.7 (88.2, 97.0)	0.907	93.9 (88.8, 97.5)	93.1 (87.8, 97.1)	0.554
BMI (Kg/m^2^)	26.05 ± 3.09	26.15 ± 2.95	0.852	26.51 (24.31, 27.76)	26.03 (23.69, 28.02)	0.487
Duration of diabetes (Year)	10.0 (5.0, 12.5)	10.0 (5.0, 20.0)	0.148	10 (5, 14)	10 (5, 20)	0.457
SBP (mmHg)	130.0 (120.0, 140.5)	140.0 (123.5, 146.0)	0.082	133.0 (121.0, 149.0)	134.0 (120.0, 143.5)	0.918
DBP (mmHg)	80.0 (70.0, 81.5)	76.0 (70.0, 84.0)	0.870	80.0 (70.0, 81.3)	75.0 (70.0, 84.0)	0.361
FBG (mmol/L)	7.7 (6.0, 9.4)	7.7 (5.8, 10.1)	0.655	7.4 (5.8, 9.3)	8.3 (6.4, 10.8)	0.434
HbA1c (%)	7.7 (6.8, 8.7)	7.7 (6.9, 8.9)	0.739	7.7 (6.9, 8.9)	7.5 (6.7, 8.4)	**0.021**
TC (mmol/L)	3.67 (3.01, 4.66)	3.85 (3.15, 4.72)	0.629	3.61 (3.00, 4.54)	3.93 (3.19, 4.77)	0.240
TG (mmol/L)	1.70 (1.25, 2.55)	1.52 (1.12, 2.09)	**0.040**	1.63 (1.16, 2.53)	1.57 (1.12, 2.08)	**0.035**
HDL-C (mmol/L)	0.91 (0.78, 1.07)	0.97 (0.84, 1.13)	**0.042**	0.90 (0.78, 1.06)	0.99 (0.88, 1.17)	** < 0.001**
LDL-C (mmol/L)	2.29 (1.63, 3.24)	2.40 (1.73, 3.19)	0.614	2.23 (1.58, 3.02)	2.50 (1.80, 3.30)	0.173
ALT (μ/L)	22.0 (13.7, 35.0)	21.6 (14.8, 32.6)	0.762	18.0 (13.2, 29.0)	29.9 (16.9, 39.4)	** < 0.001**
AST (μ/L)	18.0 (14.1, 26.5)	25.0 (17.0, 109.5)	** < 0.001**	17.0 (13.6, 22.8)	42.7 (20.4, 148.2)	** < 0.001**
Total protein (g/L)	66.2 (63.2, 69.7)	64.6 (60.3, 67.7)	**0.001**	66.1 (62.9, 69.9)	64.8 (61.0, 67.5)	**0.025**
Albumin (g/L)	42.0 (40.0, 46.0)	40.0 (38.0, 42.9)	** < 0.001**	42.0 (39.9, 44.9)	40.0 (38.0, 43.0)	** < 0.001**
γ-GT (μ/L)	28 (19, 41)	23 (19, 36)	**0.046**	25 (19, 37)	26 (20, 39)	0.440
WBC (× 10^9^/L)	7.07 (5.98, 8.39)	7.25 (5.53, 9.31)	0.600	7.03 (5.93, 8.23)	7.61 (5.45, 10.25)	0.148
Neutrophil (%)	63.33 ± 9.02	67.65 ± 11.46	**0.002**	63.40 (57.60, 68.30)	70.80 (62.35, 77.90)	** < 0.001**
Neutrophil (× 10^9^/L)	4.59 (3.53, 5.62)	4.74 (3.24, 7.23)	0.162	4.43 (3.49, 5.59)	5.39 (3.13, 7.96)	**0.002**
Lymphocyte (%)	25.8 (20.6, 32.3)	20.9 (14.8, 29.2)	** < 0.001**	26.1 (20.7, 32.3)	19.1(13.7, 27.1)	** < 0.001**
Lymphocyte (× 10^9^/L)	1.75 (1.33, 2.27)	1.50 (1.15, 1.93)	** < 0.001**	1.78 (1.37, 2.24)	1.45 (1.11, 1.87)	** < 0.001**
Neutrophil/lymphocyte	2.48 (1.79, 3.37)	3.26 (2.00, 5.18)	** < 0.001**	2.45 (1.76, 3.32)	3.73 (2.32, 5.55)	** < 0.001**
PLT (× 10^9^/L)	222.0 (183.5, 257.5)	172.0 (151.0, 207.5)	** < 0.001**	206.5 (175.5, 250.5)	170.0 (141.5, 212.5)	** < 0.001**
RBC (× 10^12^/L)	4.59 ± 0.56	4.30 ± 0.56	** < 0.001**	4.53 ± 0.54	4.31 ± 0.62	**0.014**
HGB (g/L)	138.6 ± 15.6	131.7 ± 18.0	**0.006**	136.0 (124.0, 148.0)	133.0 (122.0, 147.5)	0.310
TBIL (μmol/L)	9.4 (7.1, 12.3)	11.2 (7.9, 14.1)	**0.045**	9.3 (7.1, 11.7)	12.1 (8.5, 16.5)	** < 0.001**
DBIL (μmol/L)	3.8 (3.1, 4.9)	4.3 (3.2, 5.6)	**0.024**	3.7 (3.1, 4.8)	4.8 (3.7, 6.2)	** < 0.001**
Scr (μmol/L)	75 (62, 91)	78 (65, 99)	0.099	75 (62, 96)	78 (65, 96)	0.249
UA (umol/L)	362.0 (270.0, 409.5)	347.0 (283.5, 423.0)	0.554	357.3 ± 101.3	348.0 ± 112.7	0.907
BUN (mmol/L)	5.4 (4.5, 7.0)	5.8 (4.8, 7.8)	**0.031**	5.7 (4.6, 7.1)	5.5 (4.6, 7.8)	0.225
cTnI (ng/ml)	0.11 (0.02, 1.27)	1.70 (0.07, 22.53)	** < 0.001**	0.08 (0.02, 0.85)	4.12 (0.24, 23.9)	** < 0.001**
eGFR (ml/min)	87.0 (71.3, 98.8)	77.0 (58.0, 92.0)	**0.002**	87.0 (70.0, 98.0)	82.0 (60.5, 92.8)	**0.018**
Gensini score	42.0 (26.5, 62.5)	55.0 (31.0, 80.0)	**0.036**	42.0 (27.0, 62.2)	58.0 (32.0, 90.0)	** < 0.001**

Bold P values represent P < 0.05

### Increased NAFLD fibrosis risk was an independent risk factor for AMI in patients with T2DM co-existent NAFLD

Logistic regression analysis was performed to determine the independent risk factors for AMI in patients with T2DM co-existent NAFLD. When stratified according to NFS index, both NAFLD fibrosis intermediate risk and high risk were independent risk factors for AMI (OR = 2.663; 95% CI 1.531 to 4.630; OR = 6.388; 95% CI 3.618 to 11.277, respectively) after adjusting for sex, artery hypertension, smoking, waist circumference and eGFR. When stratified according to FIB-4 index and after adjusting for all other variables, NAFLD fibrosis high risk (OR = 6.508; 95% CI 4.316 to 9.813; p < 0.001) was an independent risk factors for AMI development (Table 3).

**Table 3:** Multivariate logistic regression analysis of NAFLD fibrosis risk for acute myocardial infarction

	OR (95% CI)	P value		OR (95% CI)	P value
NFS			FIB-4		
Low risk	Ref		Low risk	Ref	
Intermediate risk	2.663 (1.531, 4.630)	** < 0.001**	Intermediate risk	1.461 (1.000, 2.134)	0.050
High risk	6.388 (3.618, 11.277)	** < 0.001**	High risk	6.508 (4.316, 9.813)	** < 0.001**
Sex (male)	1.287 (0.868, 1.909)	0.209	Sex	1.401 (0.936, 2.099)	0.102
Artery hypertension	2.224 (1.605, 3.082)	** < 0.001**	Artery hypertension	2.292 (1.642, 3.199)	** < 0.001**
Smoking	2.027 (1.406, 2.923)	**0.043**	Smoking	2.089 (1.440, 3.030)	** < 0.001**
Waist circumference	1.869 (0.991, 3.526)	0.053	Waist circumference	1.981 (1.052, 3.731)	**0.034**
eGFR	0.369 (0.267, 0.510)	** < 0.001**	eGFR	0.326 (0.235, 0.453)	** < 0.001**

Adjusted for Sex, Artery hypertension, Smoking, Waist circumference and eGFR

Bold P values represent P < 0.05

*Ref* Reference

### Hepatic expression of FSTL3 was increased in patients with T2DM and NAFLD with fibrosis

**Fig. 2:**
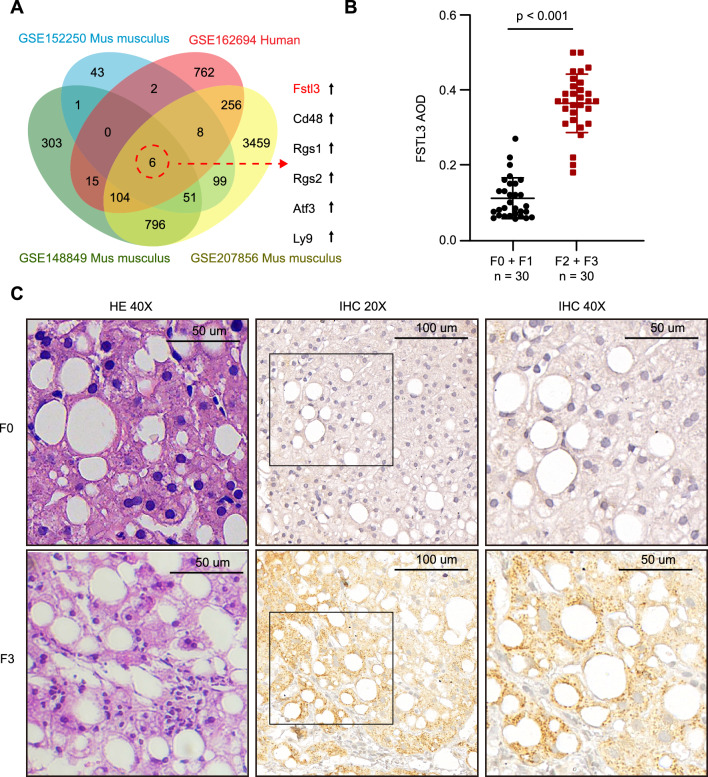
Hepatic expression of FSTL3 was increased in patients with T2DM and NAFLD with fibrosis. **A**: GEO database analysis showed FSTL3 expression in the liver of NAFLD fibrosis patients was increased. **B**–**C**: H&E staining and immunohistochemical staining of FSTL3 in liver biopsy specimens from 30 NAFLD patients with significant and advanced fibrosis (F2 and F3) and 30 NAFLD patients with fibrosis stage F0 and F1

To investigate the potential link between NAFLD fibrosis and occurrence of AMI, four RNA-seq datasets for livers with NAFLD and fibrosis/cirrhosis were obtained from the GEO database: 1 human case (GSE162694) [27] and 3 mouse cases (GSE152250 [28], GSE148849 [29], GSE207856 [30]) (Fig. 2A). To identify the specific genes involved in NAFLD fibrosis, we focused on the differentially expressed genes (> 2.5 fold change, p < 0.05) that showed significant changes between the fibrosis group and control group in four datasets. After overlapping, only 6 genes were identified: Fstl3, Cd48, regulator of G protein signaling 1 (Rgs1), regulator of G protein signaling 2 (Rgs2), activating transcription factor 3 (Atf3) and lymphocyte antigen 9 (Ly9). The expression of these six genes was up-regulated, among which FSTL3 and CD48 are secretory proteins, while the others are non-secretory membrane proteins. In addition, FSTL3 has also been shown to be closely associated with the occurrence and development of AMI [21, 22].

Immunohistochemical staining of FSTL3 was also performed on liver biopsy specimens from 30 NAFLD patients with significant and advanced fibrosis (F2 and F3) and 30 NAFLD patients with fibrosis stage F0 and F1 (Fig. 2B, C). Both significant and advanced NAFLD fibrosis patients had far more FSTL3-positive area shown by AOD [0.37 (0.32, 0.41) vs 0.09 (0.07, 0.15), p < 0.001] than patients with F0 and F1 fibrosis. Typical histological images of both hematoxylin–eosin (H&E) staining and immunohistochemical (IHC) staining with FSTL3 are presented in Fig. 2C.

### Serum FSTL3 was closely associated with AMI and NAFLD fibrosis in patients with T2DM co-existent NAFLD

**Fig. 3:**
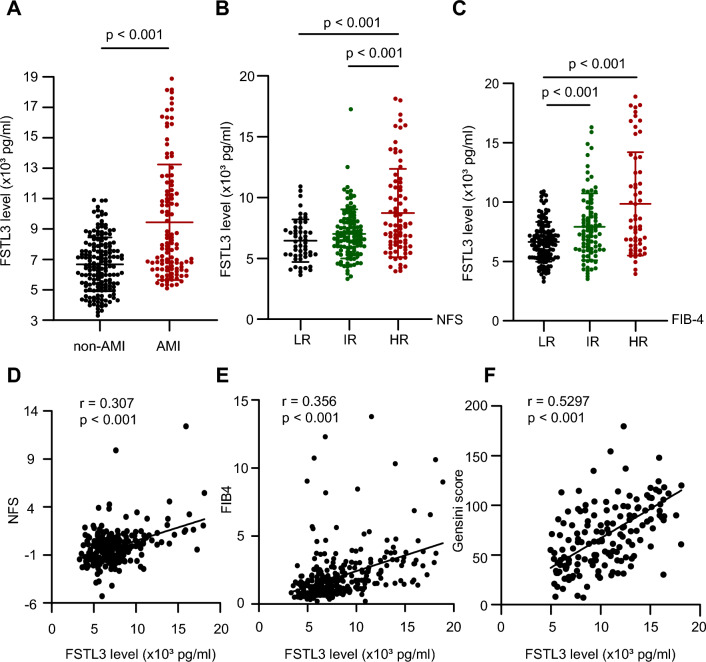
Serum FSTL3 was significantly increased in high NAFLD fibrosis risk and AMI patients, and closely associated with NAFLD fibrosis and AMI severity in patients with T2DM and co-existent NAFLD. **A**: Serum FSTL3 level in AMI patients with co-existent T2DM and NAFLD patients was significantly higher than in those without AMI. **B**–**C**: Serum FSTL3 level was much higher in NAFLD fibrosis high risk than those with NAFLD fibrosis low and intermediate risk, both by NFS and FIB-4. **D**–**F**: Serum FSTL3 was closely associated with NAFLD fibrosis and AMI severity in patients with T2DM and NAFLD

To investigate the potential mediator of the liver-heart axis, we measured serum FSTL3 of 323 T2DM and co-existent NAFLD patients (132 had AMI, and another 191 without AMI served as controls) who had serum samples in 995 T2DM co-existent NAFLD patients. As shown in Fig. 3A, serum FSTL3 level in patients with AMI was significantly higher than in those without AMI among patients with co-existent T2DM and NAFLD (7950.44 [6369.34 to 11,551.23] vs 6585.82 [5278.60 to 7933.12], p < 0.001) (Additional file 1: Table S4). After matching for age and BMI, serum FSTL3 level in AMI patients remained significantly higher than that in the non-AMI group (7713.23 [6339.19 to 11,441.58] vs 6314.38 [4992.49 to 7880.49], p < 0.001) (Additional file 1: Table S4 and Fig. 3A). Spearman correlation analysis showed that serum FSTL3 level was significantly and positively correlated with Gensini score (Fig. 3F and Additional file 1: Table S6).

Likewise, serum FSTL3 level was much higher in NAFLD fibrosis high risk patients than in those with NAFLD fibrosis low and intermediate risk, both by NFS and FIB-4 (Fig. 3B, C and Additional file 1: Table S5). More importantly, spearman correlation analysis showed that serum FSTL3 level was significantly and positively correlated with NFS, FIB-4 index (Fig. 3D, E, and Additional file 1: Table S6).

### Increased serum FSTL3 partially mediated the association of increased NAFLD fibrosis risk with AMI in T2DM patients and co-existent NAFLD

**Fig. 4:**
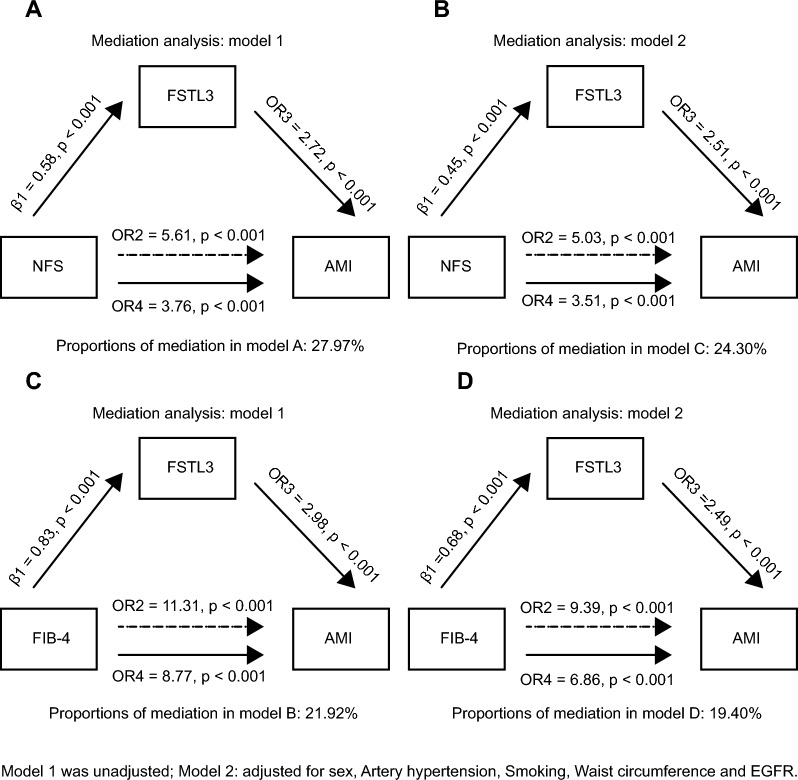
Serum FSTL3 partially mediated the association of increased fibrosis risk and AMI in NAFLD and T2DM patients. **A**, **C**: When stratified according to NFS/FIB-4, and before adjusting for other relevant variables, the mediating effect of serum FSTL3 on association of increased fibrosis risk with AMI. **B**, **D**: When stratified according to NFS/FIB-4, and after adjusting for sex, artery hypertension, smoking, waist circumference and eGFR, the mediating effect of serum FSTL3 on association of increased fibrosis risk with AMI

323 patients with T2DM and co-existent NAFLD who had serum samples were categorized by NAFLD fibrosis risk with or without AMI (191 without AMI and 132 with AMI). After standardizing the serum FSTL3 level, we performed mediation analysis of serum FSTL3 level and AMI (Additional file 1: Table S7). We found that high NAFLD fibrosis risk (stratified both by NFS and FIB-4) was an independent risk factor for AMI without adjusting for confounding factors (OR = 5.61; 95% CI 3.27 to 9.79; OR = 11.31; 95% CI 6.00 to 22.77, respectively), and high NAFLD fibrosis risk remained an independent risk factor for AMI after adjusting for sex, artery hypertension, smoking, waist circumference and eGFR (OR = 5.03; 95% CI 2.77 to 9.36; OR = 9.39, 95% CI 4.51 to 20.84, respectively). Finally, serum FSTL3 partially mediated the association of increased NFS fibrosis risk and AMI in NAFLD and T2DM patients without adjusting for other factors, and explained 27.97% of the association (Fig. 4A). After adjusting for sex, artery hypertension, smoking, waist circumference and eGFR, serum FSTL3 level explained 24.30% of the association (Fig. 4B). Serum FSTL3 partially mediated the association of increased FIB-4 fibrosis risk with AMI in NAFLD and T2DM patients without adjusting for other factors, and explained 21.92% of the association (Fig. 4C). After adjusting for all confounding factors, serum FSTL3 level explained 19.40% of the association (Fig. 4D).

## Discussion

To date, a number of studies have explored the association between NAFLD and CAD, however, this is the first one to combine noninvasive fibrosis risk indicators, NFS and FIB-4, to study the association of NAFLD liver fibrosis with AMI in T2DM co-existent NAFLD patients with large sample size. Then, GEO database analysis and immunohistochemical staining demonstrated overexpression of hepatic FSTL3 as NAFLD fibrosis progressed. Finally, the mediating role of serum FSTL3 in the association of increased fibrosis risk degree with AMI in T2DM patients with co-existent NAFLD was explored.

We first found NAFLD did not increase the risk of AMI in T2DM patients. Subsequently, using two noninvasive hepatic fibrosis indices NFS and FIB-4 (verified to be best in T2DM patients) to evaluate fibrosis risk, we confirmed the association of increased liver fibrosis with the occurrence and severity of AMI. Increased NAFLD fibrosis was an independent risk for AMI in T2DM patients. These results also demonstrated that NAFLD fibrosis was the strongest factor underlying cardiovascular events in T2DM patients with co-existent NAFLD.

GEO database analysis and semi-quantitative analysis of immunohistochemistry data showed that hepatic FSTL3 expression was enriched in the liver of patients with significant and advanced fibrosis. Elevated serum FSTL3 was verified in AMI patients with co-existent T2DM and NAFLD, and consistent with the results of previous studies in atherosclerosis patients and AMI animal models [21, 22]. Finally, this is the first study to demonstrate that serum FSTL3 level partially mediates the association of increased NAFLD fibrosis risk and AMI in patients with T2DM and co-existent NAFLD. We propose a liver-heart axis that may affect the occurrence and development of AMI. Simply described, liver fibrosis will increase serum FSTL3, and FSTL3 will reach the coronary artery through the circulation and affect the prevalence and development of AMI.

NAFLD, a hepatic manifestation of metabolic syndrome, has similar cardiometabolic risk factors to AMI, and there are several hypothesized mechanisms linking the two: 1) The association of NAFLD with other factors associated with obesity, T2DM and metabolic syndrome [31, 32] such as lipid metabolism disorder, inflammation, insulin resistance, adipokine abnormalities [33] in which mammalian target of rapamycin (mTOR) may play an important role in linking NAFLD and CAD [34], 2) genetic factors, oxidative stress, intestinal microbial disorders, adipokine and cytokine and increased activity of serum liver enzymes [35, 36]. Our study provides another possible mechanism that links NAFLD fibrosis risk with the occurrence and development of AMI. Previous studies have partially clarified the possible relationship between FSTL3 and coronary atherosclerosis and AMI. For example, it has been shown that the expression of Fstl3 is up-regulated in the heart of an AMI animal model, and induction of FSTL3 inhibits the cardioprotective effect of activin A, thus increasing sensitivity of the myocardium to ischemia [22]. Another study proved that up-regulation of FSTL3 in the serum of patients with coronary atherosclerosis could induce lipid accumulation and an inflammatory response in macrophages, thus promoting the occurrence and progression of atherosclerosis [21]. It is possible that elevated circulating FSTL3 derived from the liver of patients with hepatic fibrosis mediates the pro-atherogenic effect in T2DM. It is also acknowledged that a mechanistic insight into the potentially pathophysiological role of FSTL3 linking NAFLD fibrosis and AMI is lacking in this clinical study. Further studies using reliable rodent models to clarify the mediating function of FSTL3 are warranted.

The following limitations should be acknowledged: this study did not include patients with only NAFLD so it was not possible to analyze the association of liver fibrosis with occurrence and development of AMI in patients with only NAFLD. It was not able to determine whether serum FSTL3 played a mediating role in it, nor to verify whether T2DM and NAFLD had an additive effect on the occurrence and development of AMI.

In conclusion, this study demonstrated that hepatic fibrosis was an independent risk factor for AMI in T2DM patients, and was correlated with the severity of AMI. Increased FSTL3 expression in the liver of NAFLD fibrosis patients was also confirmed. Most importantly, we found that serum FSTL3 partially mediated the association of increased liver fibrosis risk with AMI in T2DM patients.

### Supplementary Information

**Additional file 1: Table S1.** Comparison of parameters between non-NAFLD and NAFLD patients with T2DM. **Table S2.** Multivariate logistic regression analysis of NAFLD for acute myocardial infarction. **Table S3.** Factors associated with NFS and FIB4 in patients with NAFLD co-existent T2DM. **Table S4.** Comparison of parameters between non-AMI and AMI patients with NAFLD co-existent T2DM. **Table S5.** Comparison of parameters among different NAFLD fibrosis risk stages stratified according to NFS and FIB-4. **Table S6.** Factors associated with FSTL3 in patients with NAFLD and co-existent T2DM. **Table S7.** Mediation analysis of FSTL3 in the association of increased NAFLD fibrosis risk and AMI in T2DM co-existent NAFLD patients.

## Data Availability

The datasets used and/or analysed during the current study are available from the corresponding author on reasonable request.

## Abbreviations

NAFLD: Nonalcoholic fatty liver disease
NAFL: Nonalcoholic fatty liver
NASH: Nonalcoholic steatohepatitis
GEO: Gene expression omnibus
CAD: Coronary artery disease
AMI: Acute myocardial infarction
T2DM: Type 2 diabetes mellitus
MetS: Metabolic syndrome
LSM: Liver stiffness measurement
SBP: Systolic blood pressure
DBP: Diastolic blood pressure
TGF-β: Transforming growth factor β
FSTL3: Follistatin-like protein 3
BMI: Body mass index
FBG: Fasting blood glucose
HBA_1_C: Glycated hemoglobin
TC: Total cholesterol
TG: Triglycerides
HDL-C: High density lipoprotein cholesterol
LDL-C: Low density lipoprotein cholesterol
ALT: Alanine aminotransferase
AST: Aspartate aminotransferase
TP: Total protein
ALB: Albumin
GLB: Globulin
γ-GT: Glutamine transpeptide
RBC: Red blood cell count
WBC: White blood cell count
PLT: Platelets
HGB: Hemoglobin
TBIL: Total bilirubin
DBIL: Direct bilirubin
Scr: Serum creatinine
UA: Uric acid
BUN: Blood urea nitrogen
CRP: C reactive protein
cTnI: Cardiac troponin I
eGFR: Estimated glomerular filtration rate
ELISA: Enzyme-linked immunosorbent assay
AOD: Average optical density
H&E: Hematoxylin–eosin
IHC: Immunohistochemical
NFS: NAFLD fibrosis score
FIB-4: Fibrosis index based on 4 factors
mTOR: Mammalian target of rapamycin
Rgs1: Regulator of G protein signaling 1
Rgs2: Regulator of G protein signaling 2
Atf3: Activating transcription factor 3
Ly9: Lymphocyte antigen 9
Ref: Reference
