# Supplementary material for: FSTL3 partially mediates the association of increased nonalcoholic fatty liver disease fibrosis risk with acute myocardial infarction in patients with type 2 diabetes mellitus
Source: Cardiovasc Diabetol. 2023 Oct 30;22:297. doi: 10.1186/s12933-023-02024-x (PMC10617048; doi:10.1186/s12933-023-02024-x)
Supplement: Supplementary file 1 — Additional file 1: Table S1. Comparison of parameters between non-NAFLD and NAFLD patients with T2DM. Table S2. Multivariate logistic regression analysis of NAFLD for acute myocardial infarction. Table S3. Factors associated with NFS and FIB4 in patients with NAFLD co-existent T2DM. Table S4. Comparison of parameters between non-AMI and AMI patients with NAFLD co-existent T2DM. Table S5. Comparison of parameters among different NAFLD fibrosis risk stages stratified according to NFS and FIB-4. Table S6. Factors associated with FSTL3 in patients with NAFLD and co-existent T2DM. Table S7. Mediation analysis of FSTL3 in the association of increased NAFLD fibrosis risk and AMI in T2DM co-existent NAFLD patients. [file 12933_2023_2024_MOESM1_ESM.docx]

**Table 1.** Comparison of parameters between non - NAFLD and NAFLD patients with T2DM.

|  | **Total participates (n = 1424)** | | | |
| --- | --- | --- | --- | --- |
|  | **T2DM** | **T2DM co-existent NAFLD** | | **p value** |
| N (%) | 429 (30.13%) | | 995 (69.87%) |  |
| Gender, male (n/%) | 262 (61.1%) | | 611(61.4%) | 0.905 |
| Age (Year) | 65 (58, 74) | | 62 (53, 70) | **< 0.001** |
| Smoking (n/%) | 193 (45.0%) | | 423 (42.5%) | 0.387 |
| Artery hypertension (n/%) | 188 (43.8%) | | 549 (55.2%) | **< 0.001** |
| waist circumference (cm) | 88.35 (85.00, 96.78) | | 92.89 (86.78, 101.87) | **< 0.001** |
| BMI (Kg/m^2^) | 23.62 (21.79, 24.97) | | 26.49 (24.39, 28.70) | **< 0.001** |
| Duration of diabetes (Year) | 10 (3, 17) | | 9 (4, 14) | 0.676 |
| SBP (mmHg) | 130 (120, 144) | | 131 (120, 142) | 0.159 |
| DBP (mmHg) | 78 (70, 82) | | 80 (70, 87) | **< 0.001** |
| FBG (mmol/L) | 7.21 (5.64, 9.89) | | 7.80 (6.00, 10.10) | **0.005** |
| HbA_1_C (%) | 8.30 (7.20, 10.30) | | 8.40 (7.13, 10.20) | 0.932 |
| TC (mmol/L) | 3.89 (3.14, 4.69) | | 4.25 (3.48, 5.09) | **< 0.001** |
| TG (mmol/L) | 1.19 (0.86, 1.69) | | 1.68 (1.21, 2.61) | **< 0.001** |
| HDL-C (mmol/L) | 1.04 (0.86, 1.29) | | 0.96 (0.81, 1.15) | **< 0.001** |
| LDL-C (mmol/L) | 2.38 (1.77, 3.20) | | 2.69 (1.96, 3.46) | **< 0.001** |
| ALT (μ/L) | 16.1 (11.5, 26.5) | | 21.0 (14.9, 33.1) | **< 0.001** |
| AST (μ/L ) | 17.0 (13.6, 24.4) | | 18.6 (14.1, 29.0) | **0.002** |
| Total protein (g/L) | 64.9 (61.2, 68.9) | | 66.5 (62.9, 70.8) | **< 0.001** |
| Albumin (g/L) | 41.0 (38.2, 44.0) | | 43.0 (40.0, 46.0) | **< 0.001** |
| Prealbumin (g/L) | 0.22 (0.19, 0.25) | | 0.25 (0.21, 0.28) | **< 0.001** |
| Globulin (g/L) | 23.8 (21.7, 27.1) | | 24.4 (21.4, 27.6) | 0.840 |
| γ-GT (μ/L) | 19 (13, 33) | | 28 (20, 43) | **< 0.001** |
| WBC (×10^9^/L) | 6.19 (5.07, 7.71) | | 6.58 (5.42, 7.87) | **0.010** |
| Neutrophil (%) | 62.00 (55.50, 68.7) | | 60.6 (54.3, 66.8) | **0.015** |
| Neutrophil (×10^9^/L) | 3.77 (2.97, 4.95) | | 3.92 (3.03, 5.07) | 0.225 |
| Lymphocyte (%) | 27.7 (21.1, 33.4) | | 28.9 (23.0, 34.6) | **0.006** |
| Lymphocyte (×10^9^/L) | 1.64 (1.27, 2.08) | | 1.87 (1.45, 2.30) | **< 0.001** |
| Neutrophil/ lymphocyte | 2.24 (1.69, 3.18) | | 2.09 (1.58, 2.92) | **0.007** |
| Eosinophilic granulocyte (%) | 1.9 (1.0, 3.0) | | 2.1 (1.3, 3.2) | **0.022** |
| Basophilic granulocyte (%) | 0.3 (0.2, 0.5) | | 0.4 (0.2, 0.5) | **< 0.001** |
| Mononuclear leucocyte (%) | 7.0 (5.7, 8.5) | | 6.9 (5.6, 8.2) | 0.143 |
| PLT (×10^9^/L) | 197.0 (162.3, 239.8) | | 205.0 (168.0, 247.0) | 0.065 |
| RBC (×10^12^/L) | 4.35 ± 0.57 | | 4.57 ± 0.58 | **< 0.001** |
| HGB (g/L) | 131 (120, 143) | | 138 (126, 149) | **< 0.001** |
| TBIL (mmol/L) | 9.1 (6.8, 12.8) | | 9.8 (7.3, 13.1) | **0.016** |
| DBIL (mmol/L) | 3.6 (2.7, 5.1) | | 3.7 (3.0, 4.9) | 0.251 |
| Scr (μmol/L) | 69 (58, 88) | | 69 (57, 86) | 0.553 |
| UA (μmol/L) | 297 (238, 360) | | 324 (268, 391) | **< 0.001** |
| BUN (mmol/L) | 5.8 (4.6, 7.2) | | 5.3 (4.5, 6.6) | **0.007** |
| C reactive protein (mg/dl) | 1.0 (1.0, 3.0) | | 1.0 (1.0, 6.4) | 0.596 |
| cTnI (ng/ml) | 0.02 (0.01, 0.27) | | 0.01 (0.01, 0.10) | **0.033** |
| eGFR (ml/min) | 87 (66, 98) | | 92 (75, 103) | **< 0.001** |
| AMI (n/%) | 142 (33.1%) | | 325 (32.7%) | 0.872 |

**Table 2.** Multivariate logistic regression analysis of NAFLD for acute myocardial infarction.

|  | **Model 1** | |  | **Model 2** | |
| --- | --- | --- | --- | --- | --- |
|  | **OR (95% CI)** | **p value** |  | **OR (95% CI)** | **p value** |
| NAFLD | 0.980 (0.771, 1.247) | 0.872 | NAFLD | 0.761 (0.565, 1.025) | 0.072 |
| Sex |  |  | Sex | 1.401 (0.997, 1.970) | 0.052 |
| Artery hypertension |  |  | Artery hypertension | 2.181 (1.660, 2.866) | **< 0.001** |
| Smoking |  |  | Smoking | 1.778 (1.295, 2.441) | **< 0.001** |
| Waist circumference |  |  | Waist circumference | 1.339 (0.913, 1.964) | 0.135 |
| eGFR |  |  | eGFR | 0.267 (0.203, 0.352) | **< 0.001** |

**Model 1 was unadjusted; Model 2: adjusted for Sex, Artery hypertension, Smoking, Waist circumference and eGFR.**

**Table 3.** Factors associated with NFS and FIB4 in patients with NAFLD co-existent T2DM.

|  | **NFS** | | **FIB-4** | |
| --- | --- | --- | --- | --- |
|  | **R** | **p value** | **R** | **p value** |
| Age (Year) | 0.584 | **< 0.001** | 0.512 | **< 0.001** |
| waist circumference (cm) | 0.065 | **0.05** | -0.105 | **0.001** |
| BMI (Kg/m^2^) | 0.045 | 0.178 | -0.095 | **0.004** |
| Duration of diabetes (Year) | 0.192 | **< 0.001** | 0.154 | **< 0.001** |
| SBP (mmHg) | 0.124 | **< 0.001** | 0.101 | **0.002** |
| DBP (mmHg) | -0.136 | **< 0.001** | -0.139 | **< 0.001** |
| FBG (mmol/L) | -0.049 | 0.156 | -0.039 | 0.245 |
| HbA_1_C (%) | -0.043 | 0.209 | -0.115 | **< 0.001** |
| TC (mmol/L) | -0.211 | **< 0.001** | -0.125 | **< 0.001** |
| TG (mmol/L) | -0.145 | **< 0.001** | -0.136 | **< 0.001** |
| HDL-C (mmol/L) | 0.019 | 0.572 | 0.089 | **0.007** |
| LDL-C (mmol/L) | -0.175 | **< 0.001** | -0.104 | **0.002** |
| ALT (μ/L) | -0.147 | **< 0.001** | 0.121 | **< 0.001** |
| AST (μ/L) | 0.176 | **< 0.001** | 0.499 | **< 0.001** |
| Total protein (g/L) | -0.282 | **< 0.001** | -0.126 | **< 0.001** |
| Albumin (g/L) | -0.472 | **< 0.001** | -0.274 | **< 0.001** |
| Prealbumin (g/L) | -0.341 | **< 0.001** | -0.277 | **< 0.001** |
| Globulin (g/L) | 0.088 | 0.065 | 0.127 | **0.008** |
| γ-GT (μ/L) | -0.114 | **< 0.001** | 0.028 | 0.400 |
| WBC (×10^9^/L) | -0.044 | 0.183 | -0.040 | 0.213 |
| Neutrophil (%) | 0.226 | **< 0.001** | 0.230 | **< 0.001** |
| Neutrophil (×10^9^/L) | 0.022 | 0.500 | 0.025 | 0.439 |
| Lymphocyte (%) | -0.261 | **< 0.001** | -0.256 | **< 0.001** |
| Lymphocyte (×10^9^/L) | -0.333 | **< 0.001** | -0.339 | **< 0.001** |
| Neutrophil/Lymphocyte | 0.247 | **< 0.001** | 0.242 | **< 0.001** |
| Eosinophilic granulocyte (%) | 0.091 | 0.056 | 0.111 | **0.019** |
| Basophilic granulocyte (%) | -0.104 | **0.029** | -0.046 | 0.329 |
| Mononuclear leucocyte (%) | 0.166 | **< 0.001** | 0.143 | **0.001** |
| PLT (×10^9^/L) | -0.647 | **< 0.001** | -0.612 | **< 0.001** |
| RBC (×10^12^/L) | -0.386 | **< 0.001** | -0.324 | **< 0.001** |
| HGB (g/L) | -0.326 | **< 0.001** | -0.252 | **< 0.001** |
| TBIL (mmol/L) | 0.023 | 0.563 | 0.126 | **0.001** |
| DBIL (mmol/L) | 0.085 | **0.034** | 0.179 | **< 0.001** |
| Scr (umol/L) | 0.245 | **< 0.001** | 0.172 | **< 0.001** |
| UA (umol/L) | 0.049 | 0.143 | 0.040 | 0.223 |
| BUN (mmol/L) | 0.209 | **< 0.001** | 0.170 | **< 0.001** |
| C reactive protein (mg/dl) | -0.031 | 0.622 | -0.045 | 0.472 |
| cTnI (ng/ml) | 0.392 | **< 0.001** | 0.462 | **< 0.001** |
| eGFR (ml/min) | -0.416 | **< 0.001** | -0.351 | **< 0.001** |
| Gensini score | 0.234 | **< 0.001** | 0.287 | **< 0.001** |

Table 4. Comparison of parameters between non-AMI and AMI patients with NAFLD co-existent T2DM.

|  | **Total participates (n = 323)** | | | **Matched case-control study (n = 198) (age3 & BMI3)** | | |
| --- | --- | --- | --- | --- | --- | --- |
|  | **T2DM and NAFLD** | **T2DM and NAFLD and AMI** | **p value** | **T2DM and NAFLD** | **T2DM and NAFLD and AMI** | **p value** |
| N (n/%) | 191 (59.13%) | 132 (40.87%) |  | 99 (50%) | 99 (50%) |  |
| Gender, male (n/%) | 113 (59.16%) | 91 (68.94%) | 0.073 | 62 (62.63%) | 65 (65.66%) | 0.657 |
| Age (Year) | 58 (48.75, 64) | 69 (61.25, 75) | **< 0.001** | 65 (60, 70) | 66 (59, 70) | 0.649 |
| Smoking (n/%) | 53 (27.75%) | 59 (44.70%) | **0.002** | 23 (23.23%) | 40 (40.40%) | 0.009 |
| Artery hypertension (n/%) | 99 (51.83%) | 95 (71.97%) | **< 0.001** | 47 (47.47%) | 73 (73.74%) | **< 0.001** |
| waist circumference (cm) | 88.70 (85.62, 102.49) | 89.97 (87.87, 94.32) | 0.362 | 89.02 (85.68, 103.05) | 88.73 (87.20, 95.04) | 0.332 |
| BMI (Kg/m^2^) | 26.45 (24.36, 29.42) | 25.57 (23.12, 27.58) | **0.007** | 25.71 (24.05, 28.23) | 26.21 (23.32, 27.91) | 0.752 |
| SBP (mmHg) | 129 (117, 142) | 133 (123, 140) | **0.049** | 129 (118, 143) | 132 (122, 141) | 0.303 |
| DBP (mmHg) | 80 (70, 86) | 80 (70, 84) | 0.171 | 78 (70, 86) | 80 (70, 87) | 0.621 |
| FBG (mmol/L) | 7.82 (6.00, 10.23) | 6.89 (5.68, 9.45) | 0.073 | 7.56 (5.83, 9.63) | 7.42 (5.77, 9.48) | 0.754 |
| HbA_1_C (%) | 8.55 (7.00, 10.33) | 7.50 (6.60, 8.50) | **< 0.001** | 8.90 (7.20, 10.45) | 7.70 (6.70, 8.60) | **< 0.001** |
| TC (mmol/L) | 4.37 (3.43, 5.06) | 3.89 (3.20, 4.77) | **0.034** | 4.22 ± 1.128 | 4.10 ± 1.137 | 0.468 |
| TG (mmol/L) | 1.69 (1.23, 2.82) | 1.44 (1.10, 2.13) | **0.014** | 1.53 (1.12, 2.60) | 1.48 (1.11, 2.23) | 0.814 |
| HDL-C (mmol/L) | 0.99 (0.82, 1.17) | 0.98 (0.84, 1.21) | 0.893 | 1.01 (0.87, 1.27) | 0.96 (0.84, 1.22) | 0.112 |
| LDL-C (mmol/L) | 2.69 ± 0.97 | 2.57 ± 0.97 | 0.294 | 2.58 (1.84, 3.59) | 2.53 (1.89, 3.30) | 0.613 |
| ALT (μ/L) | 20.60 (15.95, 33.10) | 22.00 (15.00, 36.00) | 0.887 | 18.75 (12.33, 27.00) | 25.50 (16.00, 38.25) | **0.005** |
| AST (μ/L) | 17.00 (13.65, 23.51) | 22.00(16.25,47.75) | **< 0.001** | 15.00 (12.90, 21.50) | 23.00 (17.00, 58.50) | **< 0.001** |
| Albumin (g/L) | 44.00 (41.00, 48.00) | 41.00 (38.00, 44.00) | **< 0.001** | 43 (41, 47) | 41 (38, 44) | **< 0.001** |
| Total protein (g/L) | 66.70 (63.50, 70.90) | 65.00 (60.95, 67.85) | **< 0.001** | 67.00 ± 5.446 | 64.90 ± 5.685 | **0.009** |
| γ-GT (μ/L) | 28.00 (19.00, 45.00) | 29.00 (19.25, 42.75) | 0.933 | 22.00 (17.00, 35.25) | 30.50 (21.00, 49.00) | **< 0.001** |
| WBC (×10^9^/L) | 6.37 (5.23, 7.54) | 7.28 (5.81, 9.29) | **< 0.001** | 6.35 (5.34, 7.32) | 7.42 (5.77, 9.58) | **< 0.001** |
| Neutrophil (%) | 58.69 ± 8.634 | 65.73 ± 12.03 | **< 0.001** | 59.06 ± 8.583 | 64.74 ± 12.301 | **< 0.001** |
| Neutrophil (×10^9^/L) | 3.73 (2.95, 4.55) | 4.64 (3.53, 6.26) | **< 0.001** | 3.73 (3.04, 4.47) | 4.63 (3.51, 6.27) | **< 0.001** |
| Lymphocyte (%) | 30.46 ± 7.89 | 23.62 ± 9.75 | **< 0.001** | 29.60 (26.30, 34.30) | 23.60 (16.70, 32.10) | **< 0.001** |
| Lymphocyte (×10^9^/L) | 1.90 (1.50, 2.42) | 1.65 (1.17, 2.08) | **< 0.001** | 1.89 (1.42, 2.15) | 1.73 (1.24, 2.19) | 0.090 |
| Neutrophil/lymphocyte ratio | 1.96 (1.54, 2.45) | 2.87 (1.80, 4.89) | **< 0.001** | 1.99 (1.66, 2.39) | 2.72 (1.78, 4.26) | **< 0.001** |
| PLT (×10^9^/L) | 212 (168, 252) | 194 (159.25, 245.75) | 0.238 | 208 (164, 250) | 191 (159, 244) | 0.593 |
| UA (umol/L) | 328.25 ± 92.25 | 372.10 ± 118.45 | **< 0.001** | 319.01 ± 95.193 | 365.78 ± 116.863 | **0.002** |
| BUN (mmol/L) | 5.20 (4.20, 6.10) | 5.80 (4.90, 8.20) | **< 0.001** | 5.50 (4.15, 6.15) | 5.50 (4.75, 7.45) | 0.086 |
| cTnI (ng/ml) | 0.01 (0.01, 0.01) | 0.11 (0.02, 1.90) | **< 0.001** | 0.01 (0.01, 0.01) | 0.13 (0.03, 2.10) | **< 0.001** |
| eGFR (ml/min) | 97.50 (88.00, 106.00) | 86.00 (63.00, 96.00) | **< 0.001** | 98.00 (87.00, 108.00) | 85.00 (64.50, 94.50) | **< 0.001** |
| NFS | -0.68 (-1.54, 0.03) | 0.37 (-0.29, 1.46) | **< 0.001** | -0.15 (-1.33, 0.50) | 0.33 (-0.37, 1.35) | **< 0.001** |
| FIB-4 | 1.05 (0.76, 1.60) | 1.98 (1.28, 3.58) | **< 0.001** | 1.23 (0.86, 1.71) | 1.90 (1.29, 3.50) | **< 0.001** |
| FSTL3 (pg/ml) | 6585.82 (5278.60, 7933.12) | 7950.44 (6369.34, 11551.23) | **< 0.001** | 6314.38 (4992.49, 7880.49) | 7713.23 (6339.19, 11441.58) | **< 0.001** |

**Table 5.** Comparison of parameters among different NAFLD fibrosis risk stages stratified according to NFS and FIB-4.

|  |  | **Total participates (n = 323)** | | | **Total participates (n = 323)** | | | |
| --- | --- | --- | --- | --- | --- | --- | --- | --- |
|  | **NFS-LR** | **NFS-IR** | **NFS-HR** | **p value** | **FIB4-LR** | **FIB4-IR** | **FIB4-HR** | **p value** |
| N (n/%) | 80 (24.77%) | 142 (43.96%) | 101 (31.27%) |  | 150 (46.44%) | 95 (29.41%) | 78 (24.15%) |  |
| Gender, male (n/%) | 41 (51.25%) | 90 (63.38%) | 52 (51.49%) | 0.097 | 97 (64.67%) | 60 (63.16%) | 47 (60.26%) | 0.807 |
| Age (Year) | 48.00 (39.00, 58.00) | 60.50 (55.00, 65.00)^**^ | 69.00 (66.00, 77.00)^**##^ | **< 0.001** | 57.00 (47.25, 63.00) | 63.00 (58.00, 69.00)^**^ | 70.00 (66.00, 78.75) ^**##^ | **< 0.001** |
| Smoking (n/%) | 20 (25.00%) | 43 (30.28%) | 41 (40.59%) | 0.067 | 40 (26.67%) | 36 (37.89%) | 39 (50.00%) | **0.002** |
| Artery hypertension (n/%) | 32 (40.00%) | 80 (56.34%) | 66 (65.35%) | 0.003 | 82 (54.67%) | 50 (52.63%) | 52 (66.67%) | 0.132 |
| waist circumference (cm) | 88.03 (85.93, 100.38) | 89.31 (86.13, 103.05) | 89.93 (87.50, 101.43) | 0.550 | 89.22 (86.01, 103.13) | 89.33 (86.48, 100.50) | 89.14 (87.20, 93.67) | 0.994 |
| BMI (Kg/m^2^) | 25.39 (23.92, 27.89) | 26.45 (24.22, 29.10) | 25.83 (23.88, 28.91) | 0.460 | 26.54 (24.13, 29.32) | 25.91 (24.57, 28.31) | 25.06 (23.12, 27.58) | 0.215 |
| SBP (mmHg) | 127.87 ± 18.11 | 129.37 ± 17.18 | 133.96 ± 15.50 | 0.058 | 128.00 (117.25, 140.00) | 133.50 (124.25, 142.00)^*^ | 134.00 (123.00, 141.50) | **0.046** |
| DBP (mmHg) | 80.11 ± 10.83 | 78.64 ± 11.52 | 78.19 ± 12.62 | 0.622 | 80.00 (71.00, 87.75) | 80.00 (70.00, 85.00) | 75.50 (70.00, 86.25) | 0.105 |
| FBG (mmol/L) | 8.73 (6.81, 10.97) | 7.53 (5.92, 9.53) | 7.52 (5.55, 9.87)^*^ | **0.023** | 7.95 (6.12, 10.56) | 7.53 (5.82, 9.59) | 7.39 (5.65, 9.33) | 0.087 |
| HbA_1_C (%) | 8.10 (7.30, 9.80) | 8.30 (6.80, 9.70) | 7.80 (7.05, 9.90) | 0.449 | 8.40 (6.90, 9.80) | 7.90 (7.00, 9.45) | 7.60 (6.45, 9.57) | 0.085 |
| TC (mmol/L) | 4.54 (3.76,5.60) | 4.28(3.37,4.84) | 3.62(3.10,4.72)^**^ | **< 0.001** | 4.46 (3.60,5.07) | 3.81(3.27,4.93) | 3.96 (3.14,4.72)^*^ | **0.012** |
| TG (mmol/L) | 2.28 (1.45, 2.92) | 1.62 (1.24, 2.60) | 1.45 (1.03, 2.27)^**^ | **0.002** | 1.69 (1.28, 2.97) | 1.56 (1.12, 2.45) | 1.40 (1.07, 2.04)^**^ | **0.003** |
| HDL-C (mmol/L) | 1.01 (0.82, 1.17) | 0.96 (0.82, 1.16) | 0.98 (0.83, 1.19) | 0.902 | 0.97 (0.82, 1.16) | 0.98 (0.85, 1.20) | 1.00 (0.86, 1.22) | 0.558 |
| LDL-C (mmol/L) | 2.86 (2.15, 3.78) | 2.61 (1.97, 3.28) | 2.19 (1.71, 3.14)^*^ | **0.029** | 2.76 ± 0.96 | 2.55 ± 0.94 | 2.49 ± 1.00 | 0.101 |
| ALT (μ/L) | 26.70 (18.00, 39.20) | 21.40 (16.00, 39.00) | 18.40 (11.00, 31.00)^*^ | **0.009** | 19.95 (15.83, 30.50) | 20.60 (14.85, 36.00) | 27.00 (16.00, 44.75) | 0.133 |
| AST (μ/L) | 17.00 (13.70, 23.10) | 18.00 (14.15, 28.70) | 18.60 (14.00, 32.00) | 0.215 | 16.00 (13.03, 21.00) | 19.70 (14.30, 32.75)^**^ | 35.50 (19.25, 109.85)^**##^ | **< 0.001** |
| Albumin (g/L) | 46.00 (42.70, 49.00) | 43.00 (41.00, 47.00)^**^ | 41.00 (38.00, 43.00) ^**##^ | **< 0.001** | 44.00 (41.00, 47.08) | 43.00 (40.40, 47.00) | 40.00 (37.25, 43.00)^**##^ | **< 0.001** |
| Total protein (g/L) | 68.09 ± 5.37 | 66.81 ± 5.20 | 64.67 ± 5.95^**##^ | **< 0.001** | 66.66 ± 5.08 | 66.39 ± 6.56 | 64.85 ± 5.71 | 0.081 |
| γ-GT (μ/L) | 32.00 (23.00, 52.00) | 29.00 (18.50, 43.00) | 25.00 (19.00, 42.00) | 0.081 | 29.00 (20.00, 42.00) | 30.00 (19.00, 45.00) | 27.00 (19.00, 50.40) | 0.993 |
| WBC (×10^9^/L) | 7.14 (5.53, 8.09) | 6.65 (5.37, 7.42) | 6.49 (4.92, 8.50) | 0.221 | 6.89 (5.63, 7.82) | 6.61 (5.18, 7.95) | 6.49 (4.84, 9.77) | 0.556 |
| Neutrophil (%) | 57.43 ± 7.68 | 60.02 ± 8.93 | 63.31 ± 13.23^**#^ | **0.003** | 59.10 (54.13, 64.78) | 59.70 (55.10, 65.45) | 68.50 (58.65, 76.97)^**##^ | **< 0.001** |
| Neutrophil (×10^9^/L) | 4.00 (3.25, 4.78) | 3.81 (3.03, 4.63) | 3.69 (2.79, 5.50) | 0.666 | 4.02 (3.34, 4.90) | 3.65 (2.89, 4.76) | 4.38 (2.95, 6.82) | 0.123 |
| Lymphocyte (%) | 31.76 ± 6.43 | 29.04 ± 8.53 | 25.72 ± 10.38^**##^ | **< 0.001** | 29.65 (24.80, 34.58) | 28.60 (23.55, 34.00) | 20.35 (13.93, 30.28)^**##^ | **< 0.001** |
| Lymphocyte (×10^9^/L) | 2.23 ± 0.70 | 1.87 ± 0.65^**^ | 1.66 ± 0.65^**#^ | **< 0.001** | 1.99 (1.58, 2.48) | 1.72 (1.38, 2.30)^*^ | 1.41 (1.09, 1.86) ^**##^ | **< 0.001** |
| Neutrophil/lymphocyte ratio | 1.89 (1.50, 2.29) | 2.09 (1.58, 2.71) | 2.27 (1.72, 4.57)^**#^ | **0.002** | 2.01 (1.55, 2.69) | 2.07 (1.65, 2.75) | 3.33 (1.90, 5.50)^**##^ | **< 0.001** |
| PLT (×10^9^/L) | 258.00 (234.00, 303.00) | 209.00 (171.25, 245.50)^**^ | 161.00 (130.00, 188.00)^**##^ | **< 0.001** | 238.50 (209.00, 268.00) | 171.00 (149.00, 195.50)^**^ | 168.00 (128.00, 224.75)^**^ | **< 0.001** |
| UA (umol/L) | 341.80 ± 91.20 | 334.60 ± 95.00 | 353.68 ± 120.45 | 0.403 | 340.00 (277.75, 401.50) | 332.00 (260.50, 403.00) | 355.50 (278.75, 431.50) | 0.505 |
| BUN (mmol/L） | 5.10 (4.20, 5.80) | 5.30 (4.28, 6.60) | 5.70 (4.80, 7.40)^*^ | **0.011** | 5.20 (4.30, 6.23) | 5.60 (4.72, 6.98)^*^ | 5.80 (4.83, 8.28)^**^ | **0.003** |
| cTnI (ng/ml) | 0.01 (0.01, 0.01) | 0.01 (0.01, 0.01) | 0.02 (0.01, 0.53)^**##^ | **< 0.001** | 0.01 (0.01, 0.01) | 0.01 (0.01, 0.08) | 0.38 (0.02, 10.30)^**##^ | **< 0.001** |
| eGFR (ml/min) | 105.50 (93.25, 114.00) | 94.00 (80.00, 103.00) ^**^ | 86.00 (62.50, 96.00) ^**##^ | **< 0.001** | 98.50 (88.00, 108.25) | 91.00 (76.00, 100.00)^**^ | 86.00 (55.50, 96.00)^**^ | **< 0.001** |
| AMI (n/%) | 14 (17.50%) | 43 (30.28%) | 75 (74.26%) | **< 0.001** | 33 (22.00%) | 39 (41.05%) | 60 (76.92%) | **< 0.001** |
| FSTL3 (pg/ml) | 6285.18 (5255.75, 7518.38) | 6775.56 (5657.98, 8066.99) | 7669.86 (5976.00, 10848.40)^**##^ | **< 0.001** | 6553.02 (5338.91, 7537.13) | 7471.52 (5982.57, 9514.73)^**^ | 8199.60 (6168.09, 12513.82)^**^ | **< 0.001** |

*, VS NFS/FIB-4 LR: p < 0.05; **, VS NFS/FIB-4 LR: p < 0.01; #, VS NFS/FIB-4 IR: p < 0.05; ##, VS NFS/FIB-4 IR: p < 0.01.

**Table 6.** Factors associated with FSTL3 in patients with NAFLD and co-existent T2DM.

|  | **FSTL3 (pg/ml)** | |
| --- | --- | --- |
|  | **R** | **p value** |
| Age (Year) | 0.364 | **< 0.001** |
| waist circumference (cm) | 0.128 | **0.043** |
| BMI (Kg/m^2^) | 0.028 | 0.660 |
| SBP (mmHg) | 0.140 | **0.019** |
| DBP (mmHg) | -0.105 | 0.080 |
| FBG (mmol/L) | -0.090 | 0.138 |
| HbA_1_C (%) | -0.091 | 0.138 |
| TC (mmol/L) | -0.162 | **0.007** |
| TG (mmol/L) | -0.119 | **0.047** |
| HDL-C (mmol/L) | 0.012 | 0.845 |
| LDL-C (mmol/L) | -0.190 | **0.001** |
| ALT (μ/L) | -0.005 | 0.938 |
| AST (μ/L) | 0.178 | **0.003** |
| Albumin (g/L) | -0.187 | **0.002** |
| Total protein(g/L) | -0.030 | 0.617 |
| γ-GT (μ/L) | 0.008 | 0.890 |
| WBC (×10^9^/L) | 0.051 | 0.394 |
| Neutrophil (%) | 0.282 | **< 0.001** |
| Neutrophil (×10^9^/L) | 0.166 | **0.005** |
| Lymphocyte (%) | -0.294 | **< 0.001** |
| Lymphocyte (×10^9^/L) | -0.249 | **< 0.001** |
| Neutrophil/lymphocyte ratio | 0.300 | **< 0.001** |
| PLT(×10^9^/L) | -0.112 | 0.061 |
| UA (umol/L) | 0.301 | **< 0.001** |
| BUN (mmol/L) | 0.423 | **< 0.001** |
| cTnI (ng/ml) | 0.257 | **< 0.001** |
| eGFR (ml/min) | -0.443 | **< 0.001** |
| NFS | 0.300 | **< 0.001** |
| FIB-4 | 0.343 | **< 0.001** |
| Gensini score | 0.530 | **< 0.001** |

**Table 7.** Mediation analysis of FSTL3 in the association of increased NAFLD fibrosis risk and AMI in T2DM co-existent NAFLD patients.

| **NFS** | **Model 1** | | **Model 2** | |
| --- | --- | --- | --- | --- |
|  | **Value (95% CI)** | **p value** | **Value (95% CI)** | **p value** |
| Total effect | 0.37 (0.24 - 0.48) | **< 0.001** | 0.37 (0.23 - 0.51) | **< 0.001** |
| Direction effect | 0.26 (0.15 - 0.38) | **< 0.001** | 0.27 (0.15 - 0.39) | **< 0.001** |
| Indirection effect | 0.10 (0.05 - 0.16) | **< 0.001** | 0.10 (0.04 - 0.17) | **< 0.001** |
| Proportions of mediation | 27.97% (14.96% - 45.00%) | **< 0.001** | 24.30% (13.27% - 40.00%) | **< 0.001** |
| **FIB-4** | **Model 1** | | **Model 2** | |
|  | **Value (95% CI)** | **p value** | **Value (95% CI)** | **p value** |
| Total effect | 0.29 (0.17 - 0.41) | **< 0.001** | 0.44 (0.29 - 0.58) | **< 0.001** |
| Direction effect | 0.23 (0.11 - 0.35) | **< 0.001** | 0.35 (0.20 - 0.50) | **< 0.001** |
| Indirection effect | 0.06 (0.03 - 0.11) | **< 0.001** | 0.09 (0.04 - 0.15) | **< 0.001** |
| Proportions of mediation | 21.92% (9.08% - 44.00%) | **< 0.001** | 19.40% (8.17% - 36.00%) | **< 0.001** |

Model 1 was unadjusted; Model 2: adjusted for Sex, Artery hypertension, Smoking, Waist circumference and eGFR.
